# Pharmaceutical Compounds in Wastewater: Wetland Treatment as a Potential Solution

**DOI:** 10.1100/tsw.2006.287

**Published:** 2006-12-28

**Authors:** John R. White, Marco A. Belmont, Chris D. Metcalfe

**Affiliations:** ^2^ Worsfold Water Quality Centre Trent University, Peterborough, Ont. K9J 7B8, Canada; ^1^ Department of Oceanography and Coastal Sciences Wetland Biogeochemistry Institute Louisiana State University, Baton Rouge, LA 70803, USA

**Keywords:** drugs, constructed wetland, water quality, sewage

## Abstract

Pharmaceutical compounds are being released into the aquatic environment through wastewater discharge around the globe. While there is limited removal of these compounds within wastewater treatment plants, wetland treatment might prove to be an effective means to reduce the discharge of the compounds into the environment. Wetlands can promote removal of these pharmaceutical compounds through a number of mechanisms including photolysis, plant uptake, microbial degradation, and sorption to the soil. We review relevant laboratory research on these various mechanisms and provide data on the few studies that have examined wetland removal. There is a need to document the degree to which various pharmaceutical compounds are removed in full-scale treatment wetlands, as there is a paucity of data on overall pharmaceutical removal rates.

## INTRODUCTION

The presence of pharmaceutically active compounds has been an issue of increasing concern and attention over the past 15 years, as these compounds have been detected in wastewater (Table 1) and subsequently find their way into surface, ground, and drinking water in North America, Australia, and Europe[1,2,3,4,5,6,7,8]. In addition to the problem of contamination of potential drinking water sources, there is concern that antibiotics released into the environment may lead to the development of antimicrobial resistance, creating a potential health threat[9]. Also, studies have shown that exposure to the anti-inflammatory drug diclofenac has been linked to the development of visceral gout and widespread population declines of several species of Asian vultures[10,11], and has also been shown to affect kidney function in trout[12,13]. Other pharmaceuticals have been shown to impact the behavior and reproductive capacity of aquatic organisms[14,15,16].

**TABLE 1**
**Select Pharmaceutical Compounds Found in Wastewater Effluent**

| Compound | Use |
|---|---|
| Bezafibrate | Lipid regulator |
| Caffeine | Stimulant |
| Carbamazepine | Antiepileptic, psychiatric drug |
| Clofibric acid | Lipid regulator (active metabolite) |
| Cotinine | Metabolite of nicotine |
| Cyclophosphamide | Antineoplastic |
| Diclofenac | Analgesic/anti-inflammatory |
| Fenoprofen | Analgesic/anti-inflammatory |
| Fluoxetine | Psychiatric drug (Prozac) |
| Gemfibrozil | Lipid regulator |
| Ibuprofen | Analgesic/anti-inflammatory |
| Indomethacin | Analgesic/anti-inflammatory |
| Ketoprofen | Analgesic/anti-inflammatory |
| Naproxen | Analgesic/anti-inflammatory |
| Norfluoxetine | Metabolite of fluoxetine |
| Pentoxifylline | Vasodilator |
| Trimethoprim | Antibiotic |

Adapted from White et al.[45]

## PHARMACEUTICALS IN WASTEWATER

Effluents from wastewater treatment plants have been found to contain a number of drugs and drug metabolites[1,17,18,19,20], with human excretion as the primary source (Table 1). Many wastewater treatment facilities discharge directly into adjacent rivers, streams, or large lakes and despite the opportunity for dilution, these compounds have still been detected in the aquatic environment. In a U.S. nationwide reconnaissance survey from 1999–2000 conducted by the U.S. Geological Survey, 80% of the 139 streams examined contained one or more of the 95 organic wastewater contaminants (OWC) tested. These compounds included prescription and nonprescription drugs, steroids, hormones, personal care products, and products of oil use and combustion. In a more recent study, Kolpin et al.[21] observed that the concentrations of pharmaceuticals in rivers downstream of towns and cities in Iowa, USA varied considerably with the hydrological conditions, with the lowest concentrations detected during periods of high water flow due primarily to dilution.

Pharmaceutically active compounds have been found in ground and drinking water[22,23,24], suggesting that some of these compounds are relatively persistent in the aquatic environment. This suggests that current treatment technologies for wastewater and, in some cases, drinking water treatment are not sufficient to remove these potentially harmful contaminants[25,26]. The focus of late has been the development of additional treatment technologies that can remove these microcontaminants from the waste stream prior to discharge into the environment. These wastewater treatment technologies include using biofilms under oxic and anoxic conditions[27], photolysis[28], and degradation with activated sludge[27].

Several drinking water treatment methods have shown some success in reducing concentrations, including ozonation[29], treatment with UV combined with hydrogen peroxide[30], filtering through granulated activated carbon[31], and reverse osmosis and/or nanofiltration[32,33]. However, while these technologies can be used to provide a safe drinking water supply to the public, they are unlikely to be widely adopted due to the cost of implementation.

The number, concentration, and chemical complexity of the various pharmaceutically active compounds in the waste stream vary widely. Further, removal of pharmaceutically active compounds from the waste stream is difficult to predict as there are a wide variety of wastewater treatment technologies used around the world. Consequently, current sewage treatment plants have observed rates of reduction of these compounds during wastewater treatment that range from almost zero to near complete removal, depending on the compound and the treatment facility[28]. This complexity of the mixture of pharmaceuticals is likely to prevent a single treatment technology from being sufficient to remove all compounds. A recent study investigating photolysis, for example, found that under oxygenated conditions, photolysis increased for some drug compounds, but did not for others[34].

## TREATMENT CHARACTERISTICS OF WETLANDS

Constructed and natural wetlands are used around the globe to treat domestic and industrial sewage/wastewater. They have been used to treat wastewater ranging from raw sewage to tertiary-treated waste streams. A number of physical, chemical, and biological factors exist within wetlands that combine to degrade, sorb, precipitate, or otherwise remove a wide range of contaminants including nutrients, organic compounds, and biological oxygen demand. For example, higher organic soils, which develop in wetland systems due to lower rates of oxidation of organic matter, can provide a sorption medium for a wide range of contaminants and remove them from surface water. In batch experiments, low sorption was found for pharmaceutically active compounds in sandy sediments[31,35], while experiments that involve containing activated carbon or organic sediments have shown high Kd values > 100[31,36] for some pharmaceuticals including ibuprofen, suggesting strong sorption potential for organic wetland soils.

The generally high microbial biomass and activity in some wetland soils may promote degradation of pharmaceuticals. For example, wetland soil microorganisms have demonstrated the ability to degrade and detoxify pentachlorophenols[37] and pesticide compounds[38,39] under both aerobic and anaerobic soil redox conditions.

Another benefit of wetland treatment is the existence of aerobic/anaerobic interfaces at the wetland soil surface and associated with the plant rhizosphere. The close proximity of aerobic and anaerobic soil volumes provides an opportunity to reduce concentrations of different drug compounds, as some pharmaceuticals are best reduced under aerobic conditions, while removal of others is favored under anaerobic conditions. An example of this concept is a study in which using aerobic biofilms showed no appreciable reduction in clofibric acid and diclofenac, but did show a 64–70% reduction in ibuprofen, while anaerobic biofilms yielded only a 20% reduction for ibuprofen and ~30–40% reduction for clofibric acid and diclofenac[34]. Andrews et al.[40] found that some pharmaceuticals were more rapidly removed from wastewater under denitrifying conditions, while others disappeared more rapidly under nitrifying conditions.

The potential also exists for photodegradation of the pharmaceutically active compounds in surface flow wetlands. Several studies have documented photodegradation of a number of drugs in surface waters including diclofenac, naproxen, and ketoprofen[5,41]. While light can be attenuated in lakes and rivers as it passes down through the water column, treatment wetlands are generally shallow systems that reduce attenuation of light and maximize photolysis. Photolysis was found to double in oxygen-saturated water for both naproxen and propanolol[34], and in the presence of nitrate. High nitrate is typical of most wastewater streams and its presence was found to reduce the half-life of five of six drugs tested in combination with photolysis[42].

Another benefit of wetlands over other aquatic systems is the presence of rooted macrophytes. As previously mentioned, plants considerably increase the amount of aerobic/anaerobic interface present in the soil[43]. Also, the stems of the plants provide a substrate for colonization of biofilms, which have been shown in lab studies to be effective in removing some pharmaceutical compounds. As the surface water flows through the plant stands, the stems and associated biofilms can potentially remove and degrade a number of compounds[43].

While many of the properties of wetlands that might prove valuable in reducing concentrations of pharmaceutically active compounds can also be found in rivers and lakes, the structure and hydraulic retention of wetlands offer a far greater opportunity for compound removal. The high soil surface area to water depth ratio, when compared to lakes and rivers, allows far greater opportunity for the pharmaceutically active compounds to come into contact with the organic soil, soil microbial populations, and plant-associated biofilms. Therefore, a greater overall percentage of the treated wastewater is in close proximity to a number of potential treatment mechanisms as it flows through the wetland system.

## WETLAND TREATMENT OF PHARMACUETICALS

There are almost no published studies on the effects of surface flow wetland treatment on the removal of pharmaceuticals from municipal wastewater, and only one we know of that is not at the pilot scale[44]. In a subsurface flow, pilot-scale wetland with both deep and shallow water depths, Matamoros et al.[45] observed that carbamazepine was sorbed onto the gravel bed and associated organic particles, while ibuprofen was removed at twice the efficiency (81%) in the more aerobic shallow bed compared with the deeper bed (48%). Therefore, removal of a number of pharmaceutically active compounds may be enhanced by wetland design, which provides exposure of compounds to a wide range of redox conditions.

For a full-scale treatment wetland[46], the authors found that of the nine pharmaceutically active compounds found in the wastewater entering the wetland, only four were detected at the wetland outflow point. Removal efficiencies ranged from 25% for fenopren to 88.2% for cyclophosphamide[44]. The other two compounds detected and not removed by wetland treatment included cotinine and carbamazepine. Carbamazepine, which is used for epilepsy treatment, has been found to be one of the most pervasive drug compounds in the environment[5]. Concentration of the compound was not a strong predictor of wetland treatment. For example, gemfibrosil found at >300 ng $l^{-1}$ entering the wetland was not detected at the wetland effluent, while cyclophosphamide at 17 ng $l^{-1}$ was still detectable after a 28-day hydraulic retention time in the wetland at 2 ng $l^{-1}$ at the outlet[44]. To underscore the potential problem of pharmaceuticals discharged into the environment, one wastewater treatment plant discharges a total of ~27 kg $year^{-1}$ of just these nine detected pharmaceutically active compounds (there were likely many others compounds present not on the initial screening list of 17). Wetland treatment has led to a significant reduction of just over 77% of the mass of these compounds that, without wetland treatment, would be discharged into the adjacent river.

## CONCLUSION

More research is needed to look more intensively at the role of soil organic matter, microbial activity, plant uptake, and biofilms on the sorption/degradation/removal of pharmaceutically active compounds from wastewater in wetland settings. While treatment wetlands are primarily designed for removal of nutrients, metals, and pesticides, it appears promising that for some classes of pharmaceutically active compounds, wetland treatment may be very effective. Data on the performance of other treatment wetlands is sorely needed to identify and narrow the list of compounds that are not reduced significantly in wetland treatment. While it may be impractical to remove all drug compounds from our waste stream, it appears that removal by wetland treatment may be possible for many compounds, thereby allowing more directed research on removal of those more persistent compounds by other technologies at the wastewater treatment plant. Toxicological studies should also be expanded to look at the effect of a wider range of pharmaceuticals on aquatic organisms. A better understanding of wetland removal pathways of pharmaceutically active compounds will allow us to better manage treatment wetlands to maximize removal of these contaminants prior to release to the aquatic environment.
